# A unified theory of calcium alternans in ventricular myocytes

**DOI:** 10.1038/srep35625

**Published:** 2016-10-20

**Authors:** Zhilin Qu, Michael B. Liu, Michael Nivala

**Affiliations:** 1 Department of Medicine, David Geffen School of Medicine, University of California, Los Angeles, California, 90095, USA; 2 Department of Biomathematics, David Geffen School of Medicine, University of California, Los Angeles, California, 90095, USA

## Abstract

Intracellular calcium (Ca^2+^) alternans is a dynamical phenomenon in ventricular myocytes, which is linked to the genesis of lethal arrhythmias. Iterated map models of intracellular Ca^2+^ cycling dynamics in ventricular myocytes under periodic pacing have been developed to study the mechanisms of Ca^2+^ alternans. Two mechanisms of Ca^2+^ alternans have been demonstrated in these models: one relies mainly on fractional sarcoplasmic reticulum Ca^2+^ release and uptake, and the other on refractoriness and other properties of Ca^2+^ sparks. Each of the two mechanisms can partially explain the experimental observations, but both have their inconsistencies with the experimental results. Here we developed an iterated map model that is composed of two coupled iterated maps, which unifies the two mechanisms into a single cohesive mathematical framework. The unified theory can consistently explain the seemingly contradictory experimental observations and shows that the two mechanisms work synergistically to promote Ca^2+^ alternans. Predictions of the theory were examined in a physiologically-detailed spatial Ca^2+^ cycling model of ventricular myocytes.

Under normal conditions, the human heart contracts once every second or so to pump blood throughout the body. The contraction of the heart is caused by intracellular calcium (Ca^2+^) release from the internal Ca^2+^ store, sarcoplasmic reticulum (SR), which is triggered by the electrical excitation of ventricular myocytes. Action potential excitation and intracellular Ca^2+^ release are two tightly regulated processes^1^. More specifically (Fig. 1A), activation of the sodium (Na^+^) current (I_Na_) gives rise to the fast upstroke of the action potential, elevating the voltage to the plateau voltage. Then the L-type Ca^2+^ current (I_Ca,L_) is activated, which maintains the long plateau. In the meantime, potassium (K^+^) currents (I_K_) are slowly activated, which repolarize the cell back to its resting potential. The Ca^2+^ brought in by L-type Ca^2+^ channels (LCCs) triggers a large amount of Ca^2+^ release from the SR and this release activity is enhanced by Ca^2+^ released from the SR, a process called Ca^2+^-induced Ca^2+^ release. Ca^2+^ released from the SR binds with myofilament (MyoF) to cause contraction. The SR is then replenished through Ca^2+^ reuptake via the sarco/endoplasmic reticulum Ca^2+^-ATPase (SERCA) pump. The Ca^2+^ that enters the cell via LCCs is extruded from the cell via Na^+^-Ca^2+^ exchange (NCX). These pumps maintain the Ca^2+^ gradient between the intracellular and extracellular space, and the intracellular Ca^2+^ homeostasis. With the presence of the Na^+^-K^+^ (NaK) pump, the gradients and homeostasis of Na^+^ and K^+^ are also maintained. Besides the normal heart rhythm, the complex regulation of membrane excitation and Ca^2+^ cycling can lead to various nonlinear dynamics in the heart that promote cardiac arrhythmias^2^,^3^,^4^,^5^, among which alternans is the most widely studied phenomenon. Alternans is a temporally period-2 pattern (Fig. 1B), which manifests as T-wave alternans in the ECG or as pulsus alternans. T-wave alternans and pulsus alternans have been known as precursors of lethal arrhythmias for more than a century^6^,^7^.

Several mechanisms of alternans have been shown^8^, including dynamical instabilities from the electrical system^9^, the intracellular Ca^2+^ cycling system^10^,^11^,^12^,^13^,^14^, or via the coupling of the two together^15^,^16^,^17^. Since voltage and Ca^2+^ are coupled via Ca^2+^-dependent ionic currents, alternans due to the electrical system will also result in alternans in the Ca^2+^ cycling system and vice versa. For Ca^2+^ cycling instability-induced alternans, two theories have been developed^18^, each of which is supported by certain experimental evidence, but none of them can completely explain the experimental observations without inconsistencies.

The first theory, proposed by Eisner *et al*.^13^, was that the slope of the fractional Ca^2+^ release (FCR) curve is responsible for alternans, which was supported by a series of experiments from Eisner’s group^11^,^12^,^13^,^14^ and others^19^. A FCR curve is defined as a functional relation between the amount of Ca^2+^ released from the SR and the SR Ca^2+^ content right before the release occurs. This SR Ca^2+^ content is called the diastolic Ca^2+^ load (DCL) (Fig. 1B). This theory was more rigorously established in later theoretical studies^20^,^21^,^22^ in which iterated maps were used to reveal that the bifurcation to alternans is determined by the interaction of the slopes of the FCR curve and the SR Ca^2+^ uptake function. The SR Ca^2+^ uptake function is defined as the amount of Ca^2+^ uptaken by the SERCA pump as a function of the amount of Ca^2+^ released in the same beat. An instability leading to alternans occurs when the slope of the FCR function is large in combination with a reduced slope of the uptake function. Since the amount of Ca^2+^ release is solely determined by the level of DCL, this theory implies that DCL will also alternate from beat to beat during Ca^2+^ alternans. However, experiments from other groups have shown that Ca^2+^ alternans can occur without DCL alternans^23^,^24^,^25^, inconsistent with the above theory that alternans is caused by a steep FCR curve.

The second theory of Ca^2+^ alternans^26^,^27^ takes into account the effects of refractoriness of SR Ca^2+^ release and the properties of individual Ca^2+^ sparks. In a ventricular myocyte, the RyRs are clustered in the cell forming Ca^2+^ release units (CRUs) in conjunction with their proximate sarcolemmal ion channel clusters (Fig. 2A). It was estimated that a ventricular myocyte might contain 20,000 to 50,000 CRUs, forming a three-dimensional CRU network inside the cell^28^,^29^,^30^. A Ca^2+^ spark is a collective random release event of a CRU^31^, which can be triggered by Ca^2+^ from the LCCs, Ca^2+^ from a nearby spark, or occur spontaneously. In this theory, alternans arises via an instability caused by the interactions of three critical properties of the individual CRUs: *R*andomness of Ca^2+^ sparks; *R*ecruitment of a Ca^2+^ spark by its neighboring CRUs; and *R*efractoriness of the CRUs. We call it the “3R theory”. An iterated map was derived using a mean-field approach, which links the Ca^2+^ spark properties to the whole-cell Ca^2+^ dynamics. This theory can explain the experimental observations^23^,^24^,^25^ that Ca^2+^ alternans can occur without DCL alternans since DCL is not a parameter or a variable in the iterated map model. The theory was verified in simulation studies using detailed Ca^2+^ cycling models^18^,^32^. Moreover, refractoriness is required for alternans to occur, which agrees with the experimental observations^23^,^25^,^33^ that refractoriness is a key parameter for Ca^2+^ alternans. However, the 3R theory cannot explain why alternans can still occur at very slow heart rates^11^,^12^,^13^,^14^ at which the RyRs should have mostly or completely recovered by the beginning of each beat.

In a recent simulation study using a detailed Ca^2+^ cycling model of ventricular myocytes^34^, we showed evidence that the two mechanisms can occur in the same ventricular myocyte under different conditions. Namely, the 3R theory is dominantly responsible for the Ca^2+^ alternans at fast pacing rates while the steep FCR mechanism is the main mechanism responsible for alternans under reduced SERCA pump activity and at normal or slow pacing rates. However, the two mechanisms are not truly independent of each other, and how they are coupled to regulate Ca^2+^ alternans remains unknown. In this study, we developed a new iterated map model for Ca^2+^ cycling dynamics under periodic pacing, which unifies the two mechanisms into a single cohesive theoretical framework. This unified theory can consistently explain the seemly contradictory experimental observations and provide novel predictions and insights into the mechanisms of Ca^2+^ alternans in ventricular myocytes.

## Results

### Development of the iterated map model

In this section, we develop a new iterated map model describing Ca^2+^ cycling dynamics under periodic pacing based on our 3R theory and the Ca^2+^ cycling properties in ventricular myocytes. The model integrates the 3R’s of the individual sparks with the whole-cell FCR of the SR, unifying the two mechanisms of Ca^2+^ alternans into a single cohesive theoretical framework of Ca^2+^ cycling dynamics under periodic pacing in ventricular myocytes.

#### The iterated maps

In our previous studies^26^,^27^, we developed an iterated map model of Ca^2+^ cycling dynamics under periodic pacing, which links the properties of the individual CRUs (or Ca^2+^ sparks) to the whole-cell Ca^2+^ cycling dynamics. The model was derived based on the following properties of Ca^2+^ release. At any time, a CRU is in one of the three states (Fig. 2B): recovered, firing (spark), and refractory. A recovered CRU may fire spontaneously due to high SR Ca^2+^ load or be activated directly by opening of LCCs. We call these types of sparks *primary firings* (or *primary sparks*). Ca^2+^ released from a primary spark may diffuse to the neighboring CRUs and recruit the recovered ones to fire^35^. We call these types of sparks *secondary firings*. After firing, a CRU remains refractory for a certain period of time. Due to the random opening properties of LCCs and RyRs, the primary sparks are probabilistic events. We assume that the probability of a CRU to fire as a primary spark during a pacing beat is *α*. Similarly, the secondary sparks are also probabilistic events, and we assume that the probability of a firing CRU to recruit a neighbor to fire during a pacing beat is *γ*. The recovery of the RyRs and SR refilling in a CRU gives rise to spark amplitude restitution^36^,^37^ and random refractory periods^38^. Here we assume that before the next pacing beat, the probability of a CRU recovering from its firing in the previous beat is *β*. If there are a total of *N*_T_ CRUs in the system, and at the k^th^ beat, there are *N*_k_ sparks, then at the following [(k+1)^th^] beat, there are *βN*_k_ unrecovered CRUs and (*N*_T_ − *βN*_k_) recovered CRUs. The number of primary sparks in this beat is then α(*N*_T_ − *βN*_k_), and thus (1 − *α*)(*N*_T_ − *βN*_k_) recovered CRUs are available for recruitment. If a fraction *f* of these CRUs are recruited to fire, then the total number of sparks at (k+1)^th^ beat is:


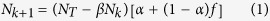


The recruitment function *f* depends on the number of sparks and the spatial pattern of the CRU states (as illustrated in Fig. 2B). As recently shown by Alvarez-Lacalle *et al*.^39^, the onset of Ca^2+^ alternans is governed by a critical phenomenon, such as in the Ising model, and it is not obvious how one can exactly calculate *f* when the system is in criticality. An approximation widely used to deal with such systems is known as mean-field approximation^40^, in which the individual random events (CRU firings in the current context) are statistically independent, i.e., the system is well mixed with no spatial patterning. We previously derived an explicit function for *f* based on such an approximation^26^,^27^, which is detailed as follows. Assume that during the (k+1)^th^ beat, a CRU has recovered from its previous firing and is available for recruitment. The probability that one of its neighbors has recovered and fires as a primary spark is 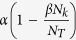. Then the probability of this CRU being recruited by the fired neighbor is 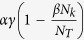, with the probability of not being recruited by this neighbor being 
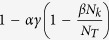
. Since there are *M* neighbors, the probability that this CRU is not recruited by any of its *M* neighbors is 
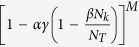
. Therefore, the total probability of this CRU being recruited by its neighbors to fire is:


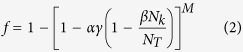


Since the recruitment of Ca^2+^ sparks is via Ca^2+^ diffusion in the cytosolic space, it depends on how fast Ca^2+^ diffuses and the distance between CRUs. CRUs farther away can be recruited to fire if the Ca^2+^ diffusion is fast or the distance between CRUs is short. Therefore, *M* can be greater than 6 in a three-dimensional cell. In this study, we used *M* = 6.

With the explicit function *f* and constants *α*, *β*, and *γ*, Eq. 1 is a closed iterated map equation, which is the governing equation of the 3R theory^26^,^27^. Eq. 1 links the properties of individual sparks to the whole-cell Ca^2+^ dynamics. The theory was verified in computer simulations using physiologically detailed Ca^2+^ cycling models^27^,^32^,^34^ and used to explain experimental observations^18^.

**Figure 1:**
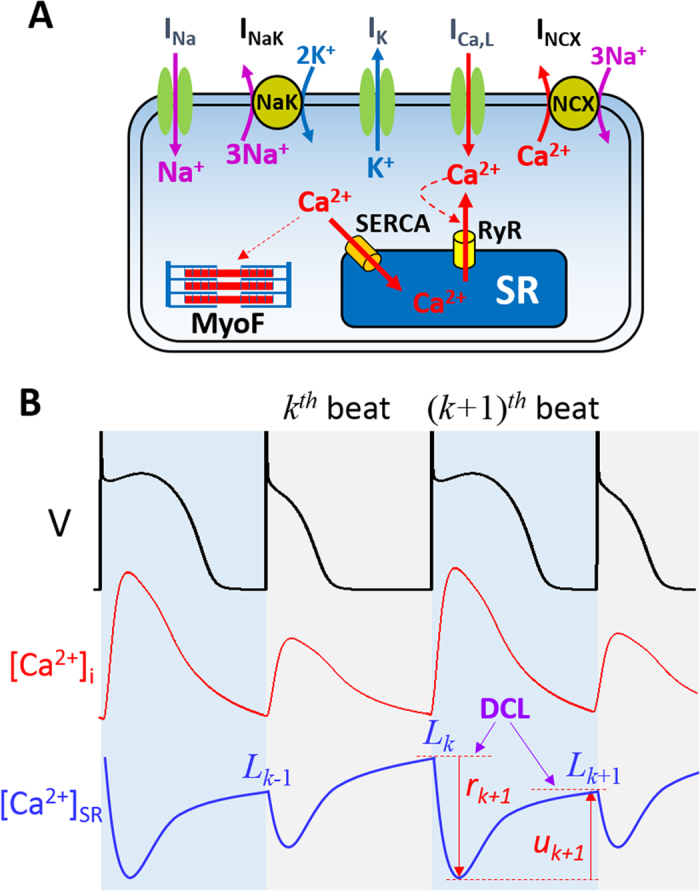
Schematic plots of excitation-coupling and alternans in ventricular myocytes. (**A**) Schematic diagram of excitation-contraction coupling in a ventricular myocyte. See text for details. (**B**) Voltage (V), whole-cell cytosolic Ca^2+^ concentration ([Ca^2+^]_i_), and whole-cell SR Ca^2+^ concentration ([Ca^2+^]_SR_) illustrating alternans under periodic pacing. *L*_k_, *r*_k+1_, and *u*_k+1_ were defined graphically.

In the original 3R theory, *α*, *β*, and *γ* were assumed to be constants. In a later study^32^, we showed that *α* and *γ* depended on DCL. However, DCL may not be a constant and can change from beat to beat during alternans. Therefore, if a varying DCL is added into the model, Eq. 1 is no longer a closed system and an additional equation is needed to describe DCL. Simply following the conservation law (as illustrated in Fig. 1B), the equation for DCL is:


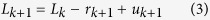


where *L*_k+1_ is the DCL of the present beat and *L*_k_ is that of the previous beat. *r*_k+1_ is the amount of Ca^2+^ released from the SR via Ca^2+^ sparks at the present beat and *u*_k+1_ is the amount of Ca^2+^ re-uptake into the SR via SERCA pump at the present beat.

Since a Ca^2+^ spark is a unitary release event, the amount of Ca^2+^ released from the SR is proportional to the total number of sparks during a pacing beat, and thus we can denote *r*_*k*+1_ as:


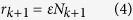


where *ε* describes the amplitude of the Ca^2+^ sparks. Note that due to randomness and heterogeneities, the amplitudes of different individual sparks in a myocyte are not necessarily the same, and ideally one would use 
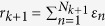
 if the full spark amplitude distribution were available. For simplicity, we assume an average spark amplitude determined by DCL and RyR recovery, whose explicit mathematical form will be detailed below.

Since the amount of Ca^2+^ re-uptake into the SR depends not only on the machinery of SERCA but also on the peak and diastolic cytosolic Ca^2+^ concentration, we denote the amount of Ca^2+^ uptaken by the SR as a function of spark number as


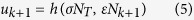


where *σ* is a parameter describing the background net Ca^2+^ uptake (or leak). An explicit form of function *h* will be derived later.

Inserting Eqs 4 and 5 into Eq. 3 leads to a more explicit form:


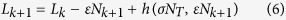


which links the number of sparks to DCL. Note that in previous studies^20^,^21^,^22^, Eq. 3 was also used in the theory of steep FCR induced Ca^2+^ alternans, in which FCR and SR Ca^2+^ uptake were described by phenomenological functions of DCL. Here we express FCR and SR Ca^2+^ uptake as functions of spark number, which then links Ca^2+^ sparks to DCL. In the old models^20^,^21^,^22^, a constant total Ca^2+^ had to be used to keep Eq. 3 in a closed form, but this condition is no longer required in the new theory. The total Ca^2+^ of the cell is determined by the equation itself, i.e., the total Ca^2+^ is a solution of the model instead of a parameter, which changes in time.

Eqs 1 and 6 are therefore the two governing equations of the iterated map model describing intracellular Ca^2+^ cycling dynamics under periodic pacing. Since *α* and *γ* are functions of DCL (*L*_k_), i.e., *α* = *α*(*L*_*k*_) and *γ* = *γ*(*L*_*k*_), Eqs 1 and 6 are two-way coupled, forming a closed set of equations describing the dynamics of both the cytosolic and SR Ca^2+^. By denoting *n*_*k*_ = *N*_*k*_/*N*_*T*_ and *l*_*k*_ = *L*_*k*_/*N*_*T*_, Eqs 1 and 6 take on the following dimensionless forms:


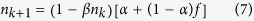



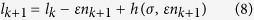


*f* becomes a function of *n*_k_ and *l*_k_, i.e.,


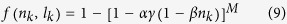


since *α* and *γ* are functions of *l*_k_, i.e., *α* = *α*(*l*_*k*_) and *γ* = *γ*(*l*_*k*_). Besides *α* and *γ*, *β* and *ε* are also functions of *l*_k_ and/or pacing period *T*. These functions, as well as function *h*, will be defined in detail in the sections below.

For the purpose of our theoretical analysis, we rewrite Eqs 7 and 8 in a more general form as


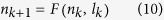



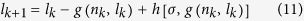


where


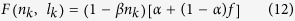


and


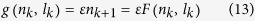


*Primary spark rate* (*α*)—It is well known that the spark probability (or frequency as traditionally used in the literature) increases with SR Ca^2+^ content^41^,^42^, however, a quantitative relation is not available experimentally. Based on the numerical simulations in our previous study^32^, *α* is a sigmoidal function of DCL (*l*_k_), i.e.,


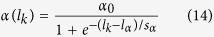


where *l*_α_ is a parameter determining the half value of function *α*(*l*_k_) and *s*_α_ is a parameter determining the slope of function *α*(*l*_k_). *α*_0_ describes the coupling fidelity between the proximate LCC cluster and the RyR cluster when the DCL is high, whose value lies between 0 and 1. *α*_0_ = 1 represents a perfect coupling between the LCC cluster and the RyR cluster, indicating that opening of the LCCs always causes the RyR cluster to fire. *α*_0_ = 0 means no coupling, indicating that opening of the LCCs always fails to cause the RyR cluster to fire. Physiologically, *α*_0_ is determined by the LCC open probability and conductance, the volume of the dyadic space, and the RyR sensitivity to Ca^2+^, etc.

*CRU refractoriness* (*β*)—The CRU refractoriness is determined by the recovery time of the RyRs. Here we simply assume that *β* only depends on the pacing period *T* in a sigmoidal function as:


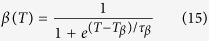


where *T*_β_ determines the half value of *β* and *τ*_β_ determines the slope of the sigmoidal function.

*Spark recruitment rate* (*γ*)—No experimental data is available for *γ*. Again based on numerical simulations^32^, *γ* is also a sigmoidal function of DCL (*l*_k_), i.e.,


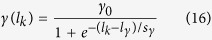


where *l*_γ_ is a parameter determining the half value of function *γ*(*l*_k_) and *s*_γ_ is a parameter determining the slope of function *γ* (*l*_k_). *γ*_0_ describes the coupling between CRUs when the DCL is high, whose value lies between 0 and 1. *γ*_0_ = 0 indicates that a Ca^2+^ spark of a CRU will never cause its neighboring CRUs to fire, while *γ*_0_ = 1 indicates that a fired Ca^2+^ spark will always cause its neighboring CRUs to fire. Physiologically, *γ*_0_ is determined by the distance between CRUs, the cytosolic Ca^2+^ diffusion rate, and the RyR sensitivity to Ca^2+^, etc.

*Spark amplitude and restitution* (*ε*)—SR Ca^2+^ release and spark amplitude restitution have been measured and characterized experimentally^36^,^37^,^38^,^43^,^44^, and is jointly determined by the recovery of the RyRs and the SR refilling. Therefore, *ε* is a function of *l*_k_ and pacing period *T*, i.e., *ε* = *ε*(*l*_*k*_, *T*). We assume the following function for *ε*:


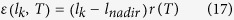


where *l*_*nadir*_ is the SR Ca^2+^ content at the peak of the spark and *r*(*T*) is the spark amplitude restitution function. We set *r*(*T*) as^36^


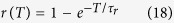


where *τ*_r_ is the time constant.

#### Fractional SR Ca^2+^ release function (g)

During a heartbeat, only a fraction of the SR free Ca^2+^ is released, which is determined by the DCL and the recovering status of the RyRs. Such a release-load relationship was first experimentally measured by Bassani *et al*.^45^ and then in more detail by Shannon *et al*.^46^. The FCR function is a nonlinearly increasing function of DCL (Fig. 3A,B). In the previous theoretical studies^19^,^21^,^22^, phenomenological functions were used for FCR. Here we provide a more mechanistic interpretation of this function in terms of spark properties. Based on Eq. 13, the FCR function *g* is a function of *n*_k_ and *l*_k_ as


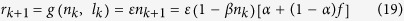


When *T*→∞, according to Eqs 15 and 18, *β* = 0 and *r* = 1, all CRUs are recovered, thus the function *g* depends solely on *l*_k_ since *α*, *γ*, and *ε* are functions of *l*_k_, i.e.,


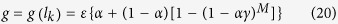


If there is no recruitment, i.e., *γ*_0_ = 0, then Eq. 20 becomes


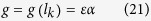


In Fig. 3A we plot the *g* functions Eq. 20 (red) and Eq. 21 (green) against the experimental data by Bassani *et al*.^45^ and in Fig. 3B the fraction (*g*/*l*_k_) against the experimental data by Shannon *et al*.^46^ for comparison. The presence of the recruitment steepens the FCR curve, which has been also demonstrated in our simulations using detailed Ca^2+^ cycling models^34^,^47^. Note that phenomenological functions were used for FCR based on the experimental measurements in previous studies^20^,^21^,^22^ without a mechanistic interpretation. Eqs 19, 20, 21 link the spark properties to FCR, providing a mechanistic interpretation of FCR.

#### SR Ca^2+^ uptake function (h)

It’s generally assumed that the SERCA pump flux obeys a Hill-type kinetics as:


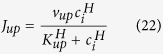


where *v*_up_ is the maximum amplitude of the SERCA pump activity, *K*_up_ is the Ca^2+^ concentration at half SERCA activity, *c*_i_ is the cytosolic Ca^2+^ concentration, and H is the Hill coefficient. Therefore, the amount of Ca^2+^ taken up by the SERCA pump depends not only on the amplitude of the cytosolic Ca^2+^ concentration but also on its time course of decay. Here we assume that *c*_i_ decays exponentially: *c*_*i*_ = *c*_*b*_ + *c*_*p*_*e*^−*λt*^, where *c*_b_ is the baseline Ca^2+^ and *c*_b_+*c*_p_ is the peak Ca^2+^. *c*_p_ is then proportional to the amount of Ca^2+^ released from the SR, i.e., *c*_*p*_ = *εn*_*k*+1_. During a single beat, the amount of Ca^2+^ pumped back into the SR can be calculated as


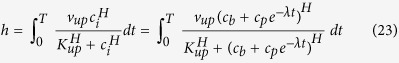


which for *H* = 1 leads to:


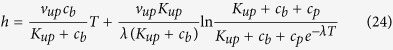


Assume that at the end of the cycle, the Ca^2+^ decays to the baseline, *c*_i _~ *c*_b_, i.e., *c*_*p*_*e*^−*λt*^ ≈ 0. Since *c*_*b*_ ≪ *c*_*p*_∝*εn*_*k+1*_, we can then simplify Eq. 24 to


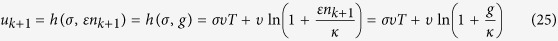


where *υ* describes the maximum SERCA pump activity and *κ* corresponds to the *K*_up_ of SERCA. The slope of this function is


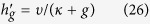


which is a decreasing function of *g*.

It was estimated *H*~1.7 for physiological cells^48^. For *H* > 1, an explicit form for the function *h* may not be possible from Eq. 23, and even when it is possible that Eq. 23 can be integrated analytically (e.g., *H* = 2), the function is too complex to be useful. However, it is generally true that *h* is an increasing function of *g* and its slope is a decreasing function of *g*, and therefore we use Eq. 25 as the uptake function for this study.

### Predictions from the iterated map model

We first performed a general linear stability analysis of the model to obtain the stability criterion. We then used numerical simulations of the iterated map model to investigate the nonlinear dynamics and make more specific theoretical predictions.

#### General linear stability analysis

The steady state of the iterated map model can be solved from the following equations, derived from Eqs 10 and 11 by setting *n*_k+1_ = *n*_k_ = *n*_ss_ and *l*_k+1_ = *l*_k_ = *l*_ss_:


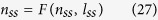



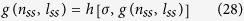


where *n*_ss_ and *l*_ss_ are the steady-state values of the number of sparks and DCL, respectively. The steady state can be graphically determined using Eq. 28, as shown in Fig. 3D. The stability of the steady state is determined by the following eigenvalues:


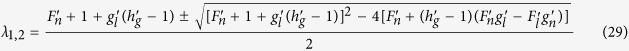


where 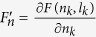, 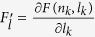, 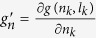, 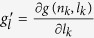, and 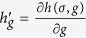, which are all partial derivatives evaluated at the steady state. These functions have the properties: 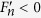, 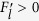 and 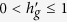, and satisfy the relationships:


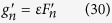


and


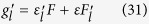


The steady state is stable when both |*λ*_1_| < 1 and |*λ*_2_| < 1, and becomes unstable when |*λ*_1_| > 1 or |*λ*_2_| > 1. Alternans occurs when *λ*_1_ < −1 or *λ*_2_ < −1. Applying this condition to Eq. 29 and using Eqs 30 and 31, one obtains the stability criterion for the steady state as


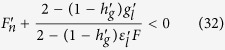


i.e., the steady state is unstable as long as Eq. 32 holds.

We first analyze two special cases:

(1) *β* = 0 and *r* = 1. *β* = 0 means no refractoriness of SR Ca^2+^ release and *r* = 1 means no spark restitution. Under this condition, 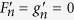, and the two maps are no longer coupled since *g* = *g*(*l*_*k*_) is solely a function of DCL (see Eq. 20). The model is then reduced to a single map (the other one becomes trivial) as


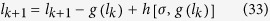


and the eigenvalues in Eq. 29 become


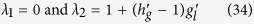


Since there is no refractoriness, the instability is solely determined by the steepness of the FCR curve and that of the SR Ca^2+^ uptake curve, independent of the 3R’s. Under this condition, alternans occurs when *λ*_2_ < −1, which leads to:


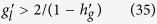


which can be also obtained from Eq. 32 by setting 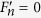. Eq. 35 indicates that the steady state tends to become unstable via increasing the slope of the FCR curve 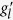 and/or reducing the slope of SR Ca^2+^ uptake function 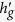. Note that this stability criterion is slightly different from the one derived in previous studies^19^,^20^,^21^,^22^,^49^, in which it was 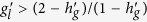. This is because previous studies assumed the total Ca^2+^ to be constant in the cell, which is not true since the cell is not a closed system for Ca^2+^. In the current model, the total amount of Ca^2+^ of the cell is not set as a parameter but a variable, and thus Eq. 35 is a more accurate stability criterion.

(2) 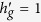. 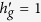 is always satisfied when *g* = *h*, i.e., the SR always reuptakes exactly the same amount of Ca^2+^ that it releases within a beat. When 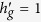, the SR refills to the same level before each beat, i.e., *l*_*k+1*_ = *l*_*k*_. This corresponds to the experiments^23^,^24^,^25^ in which the SR was refilled to the same level in each beat during alternans. Under this condition, the two map equations are also decoupled, and the stability criterion is


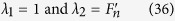


The condition for alternans is then 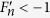, and thus the stability of the system is solely determined by the 3R’s. Figure 4A shows some examples of 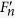. Since F is a decreasing function of *n*_k_, 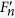 is always negative and the instability is promoted by decreasing 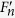 (note: decreasing 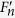 means that the slope of F becomes steeper). As shown in Eq. 1, the nonlinearity comes from the recruitment, which depends on the probability of primary sparks and refractoriness. The condition for 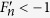 requires an intermediate *α* (not all recovered CRUs are firing so that there are still CRUs available for recruitment), a large *γ*, and a very large *β* (>0.9), as shown in Fig. 4B.

In general, the two maps are coupled, and the stability criterion of the coupled system is governed by Eq. 32. The steady state is unstable when Eq. 32 holds, which is synergistically promoted by decreasing 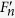, increasing 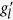, and decreasing 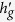, indicating that the two mechanisms help each other in promoting alternans. The synergistic effects of the two mechanisms in promoting alternans are shown in Fig. 4C,D. Specifically, when 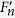 is more negative, alternans occur at a smaller 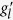 for the same 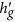 (Fig. 4C), and when 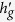 is smaller for a given 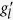, a smaller slope of *F* is needed for alternans (Fig. 4D). In the 3R theory, a very large *β* is needed to produce a steep enough *F* function for alternans (Fig. 4A,B). In the unified model, alternans can occur at a much smaller *β* value due to the synergistic effects.

#### Numerical simulations

Besides the general theoretical predictions, more specific predictions can be obtained by directly simulating the iterated map model. Depending on the choice of parameters, the model can exhibit alternans, different periodicities, as well as chaos. Alternans occurs at either fast or very slow heart rates. Here we choose the FCR function (and thus *α* and *γ*) based on the experimental FCR data by Shannon *et al*. (the red curve in Fig. 3B)^46^ and the uptake function in Fig. 3D (the black curve) as a control set of parameters. A bifurcation diagram for these control parameters is shown in Fig. 5A in which alternans occurs at fast pacing (*T* < 450 ms). Refractoriness is a key parameter that promotes this behavior.

We first studied the effects of the SERCA pump which is described by the parameters *υ* (maximum amplitude of SERCA activity, *v*_up_) and *κ* (*K*_up_ of SERCA). Reducing *υ* tends to suppress alternans at fast pacing rates but promote alternans at slower pacing rates (Fig. 5B). Reducing *κ* has a small effect on the onset of alternans and increases the alternans amplitude (Fig. 5C). Reducing both, however, promotes alternans at both fast and slow pacing rates (Fig. 5D). As shown in Fig. 3D, by reducing *υ* and *κ* properly, one can maintain the same steady state while decrease the slope of *h* at the steady state (see Fig. 3D). Based on the predictions of the general theory (Fig. 4), reducing 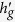 promotes both mechanisms of alternans. Reducing *υ* alone reduces 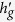, but also changes the steady state.

Increasing the slope of the FCR curve promotes the instability, causing alternans to occur at slower pacing rates and a route to chaos via a period doubling bifurcation (Fig. 5E). This instability is further enhanced by reducing 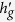 via reducing *υ* and *κ* (Fig. 5F).

To investigate the effects of spark properties on the mechanisms of alternans, we chose another set of control parameters in which strong alternans exists for both mechanisms (Fig. 6A). Reducing the primary spark probability *α* (Fig. 6B) or the recruitment strength *γ* (Fig. 6C) suppresses alternans promoted by both mechanisms. Increasing the refractory period potentiates alternans caused by fast pacing (Fig. 6D,E). In the simulations above, we chose to omit the spark amplitude restitution. Experiments from Sobie *et al*.^36^ showed time constants to be around 100 ms (i.e., *τr *~ 100 *ms*), while others^43^ showed a much longer time constant (*τ*_*r *_~ 450 *ms*). In Fig. 6F, we show bifurcation diagrams for different *τ*_r_ ( = 0 ms, 100 ms, 200 ms, and 300 ms). Adding the spark amplitude restitution into the model changed the onset of alternans, causing the FCR mediated alternans to occur at a longer *T* and the refractoriness mediated alternans to occur at a shorter *T*. For *τ*_r_ = 300 ms, the refractoriness mediated alternans is completely suppressed. Note that the magnitude of spark number alternans at fast pacing is increased for *τ*_r_ = 100 and 200 ms. Based on Eqs 17, 18, and 31, the direct consequence of reducing the spark amplitude is the reduction of 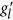, which suppresses the instability based on linear stability analysis. However, the reduction of spark amplitude also increases SR load, causing more CRUs to fire (see Fig. 6F, in which *n*_k_ increases in the non-alternans regime as *τ*_r_ increases), and thus amplifies the amplitude of spark number alternans.

### Validation of theoretical predictions using a ventricular cell model with detailed spatiotemporal Ca^2+^ cycling

**Figure 2:**
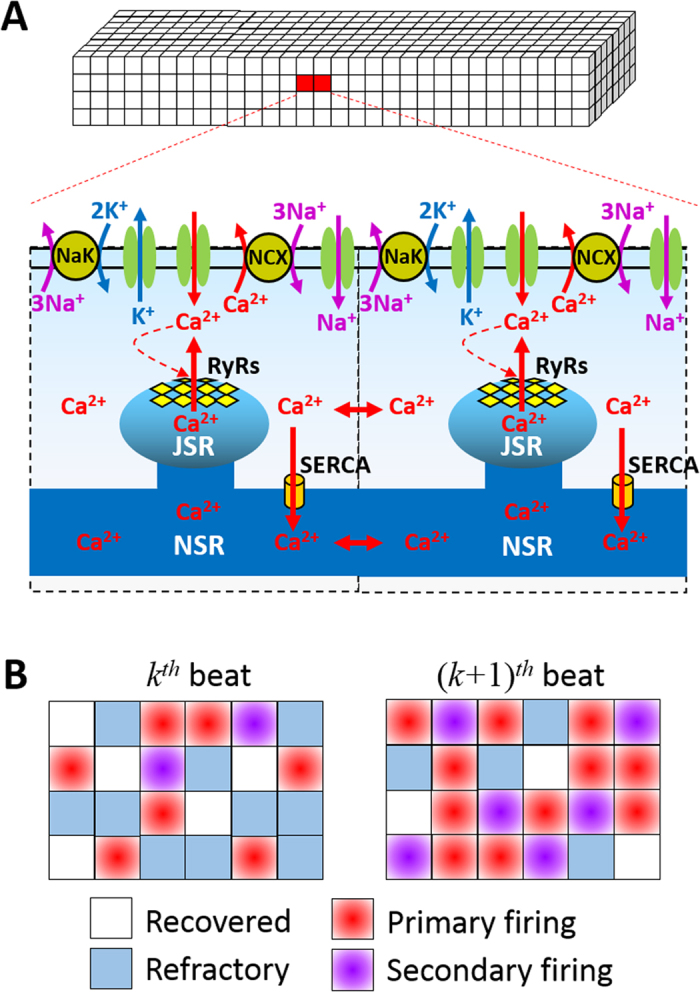
Schematic diagrams for spatiotemporal Ca^2+^ cycling in ventricular myocytes. (**A**) Schematic diagrams of CRU network representing a ventricular myocyte, excitation-contraction coupling in a CRU, and CRU coupling via Ca^2+^ diffusion. JSR—junctional SR and NSR—network SR. (**B**) Schematic CRU firing patterns.

**Figure 3:**
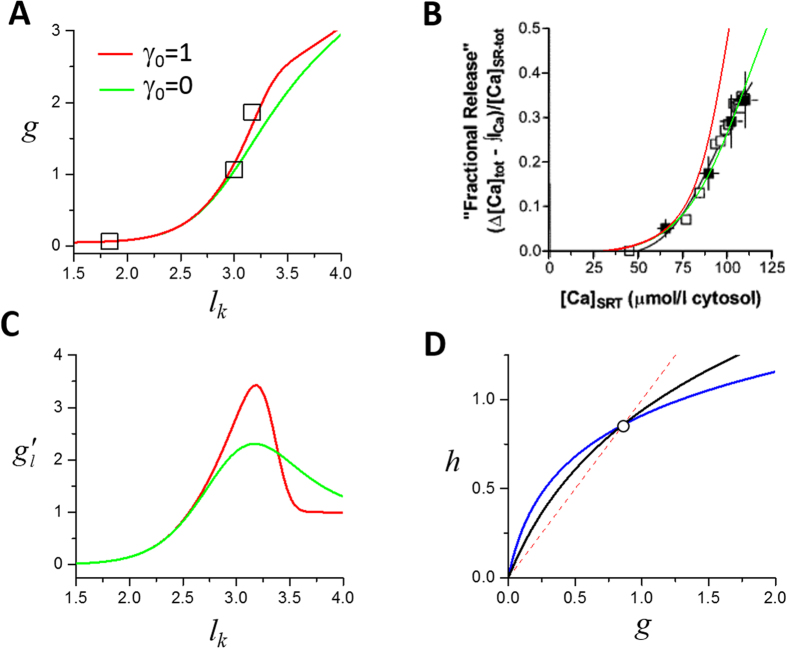
Fractional SR Ca^2+^ release and uptake functions and slopes. (**A**) The amount of Ca^2+^ released (*g*) versus SR Ca^2+^ content (*l*_k_) using Eq. 20 (red, *γ*_0_ = 1) and Eq. 21 (green, *γ*_0_ = 0). *l*_α_ = 3, *s*_α_ = 0.3, *l*_γ_ = 3.5, *s*_γ_ = 0.15, and *α*_0_ = 1 were used. Squares are data from Bassani *et al*.^45^ scaled by dividing both the SR Ca^2+^ content and the amount of Ca^2+^ released by 30. (**B**) Percentage of SR Ca^2+^ content released (*g*/*l*_k_) versus SR Ca^2+^ content using Eq. 20 (red, *γ*_0_ = 1) and Eq. 21 (green, *γ*_0_ = 0). *l*_α_ = 3.5, *s*_α_ = 0.6, *l*_γ_ = 3.5, *s*_γ_ = 0.3, and *α*_0_ = 1 were used. Symbols are the original plot of Shannon *et al*.^46^. The SR Ca^2+^ content in Eqs 20 and 21 were scaled (multiplied by 30) to match the experimental plot. (**C**) The slope of g (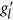) versus DCL for the two curves shown in (**A**). (**D**) The amount of Ca^2+^ uptaken by the SR (*h*) versus *g* (Eq. 25). Black line: *υ* = 0.75 and *κ* = 0.4. Blue line: *υ* = 0.38 and *κ* = 0.1. Dashed line: line of identity. The intersection of *h* with the line of identity is the steady state (circle).

To demonstrate some of the theoretical predictions from the iterated map model, we carried out computer simulations using a ventricular cell model with detailed spatiotemporal Ca^2+^ cycling. The model is a three-dimensional network of CRUs (as illustrated in Fig. 2A), consisting of 200 × 20 × 10 CRUs. The LCCs and RyRs were modeled by stochastic Markov transitions. The details of the model and numerical methods for computer simulations can be found in our previous publications^34^,^50^.

To induce Ca^2+^ alternans in the model, we used an action potential clamp protocol with an action potential waveform from the normal conditions with [Na]_i_ clamped to 12 mM (see Nivala *et al*.^34^). Figure 7A show peak [Ca^2+^]_i_ versus pacing period (*T*), showing Ca^2+^ alternans occurs at fast pacing (*T* < 450 ms). Reducing the SERCA pump amplitude *v*_up_ by 50% attenuates the alternans amplitude, and causes alternans to occur at slower pacing (*T* < 750 ms), but also tends to suppress alternans at fast pacing. In general, this agrees with the iterated map results shown in Fig. 5B. Reducing the *K*_up_ of the SERCA pump by 50% has a small effect on the onset of alternans while increasing the amplitude of alternans. Reducing both the amplitude and the *K*_up_ by 50% greatly changes the dynamics (Fig. 7D,E). First, it causes alternans to occur at much slower heart rates (*T* < 3250 ms). Second, high periodicity and irregular dynamics occur via a period-doubling bifurcation as *T* decreases. As *T* decreases even further, a stable period-1 state occurs, followed by a sudden transition to a period-3 state, and finally to irregular dynamics. This is very similar to the bifurcation sequence of the iterated map model shown in Fig. 5F. Although stochasticity exists in the detailed Ca^2+^ cycling model, the irregular dynamics is likely chaos since it only occurs for certain pacing periods and arises via period-doubling bifurcations. Moreover, the bifurcation diagram is very similar to the one obtained using the iterated map model in which the irregular dynamics is indeed chaos (Fig. 5F).

## Discussion

In this study, we developed an iterated map model describing the intracellular Ca^2+^ cycling dynamics in ventricular myocytes under periodic pacing. The model links the Ca^2+^ spark properties to FCR, providing a mechanistic interpretation of FCR. It unifies the two known mechanisms of Ca^2+^ alternans into a single theoretical framework, which shows that the two mechanisms work synergistically to promote alternans. Based on this new model, at slow heart rates where the RyRs may have already recovered at the beginning of each beat, alternans is promoted by steepening the FCR function and/or reducing the slope of the SR Ca^2+^ uptake function. CRU coupling (the *γ* factor) plays an important role in promoting this mechanism of alternans by steepening the FCR function. At fast heart rates, however, the 3R’s, the increased FCR function slope, and the reduced SR Ca^2+^ uptake function slope work synergistically to promote alternans. The new model can consistently explain the seemingly contradictory experimental observations, as detailed below.

**Figure 4:**
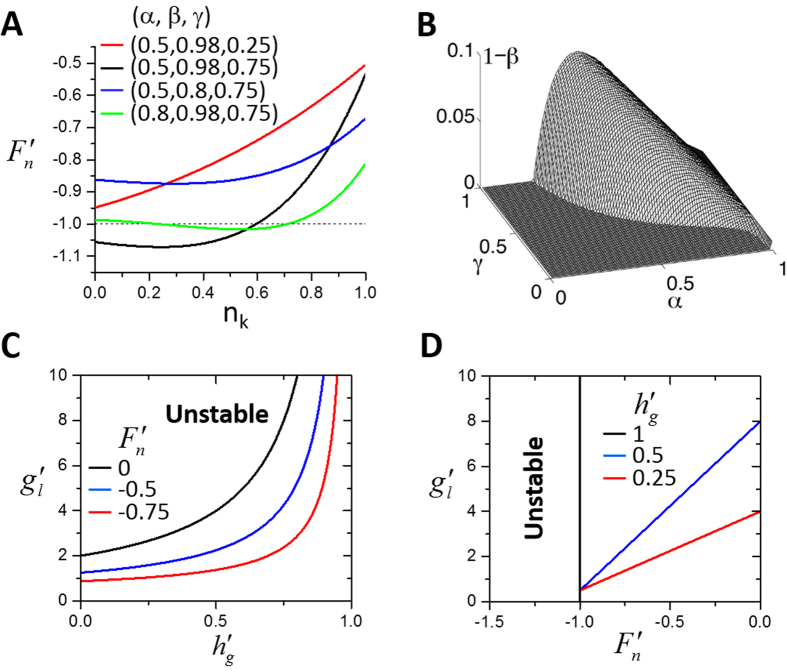
Predictions of general linear stability analysis. (**A**) The slope of function *F* (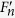) versus spark number *n*_k_ for different combinations of *α*, *β*, and *γ*. (**B**) Unstable region in *α*-*β*-*γ* space (underneath the hill). For visual purpose, 1-*β* instead of *β* was used for the vertical axis. In A and B, *α*, *β*, and *γ* were simply treated as constants as in the original 3R theory instead of functions of *l*_k_ or pacing period *T* as in the rest of the present study. (**C**) Stability boundaries determined using Eq. 32 for different 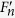 values. Black: 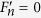; Blue: 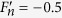; Red: 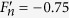. *F* = 0.5 and 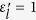 were used. The steady state is unstable above the lines. (**D**) Stability boundaries determined using Eq. 32 for different 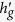 values. Black: 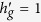; Blue: 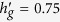; Red: 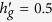. *F* = 0.5 and 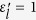 were used. The steady state is unstable above or left to the lines.

**Figure 5:**
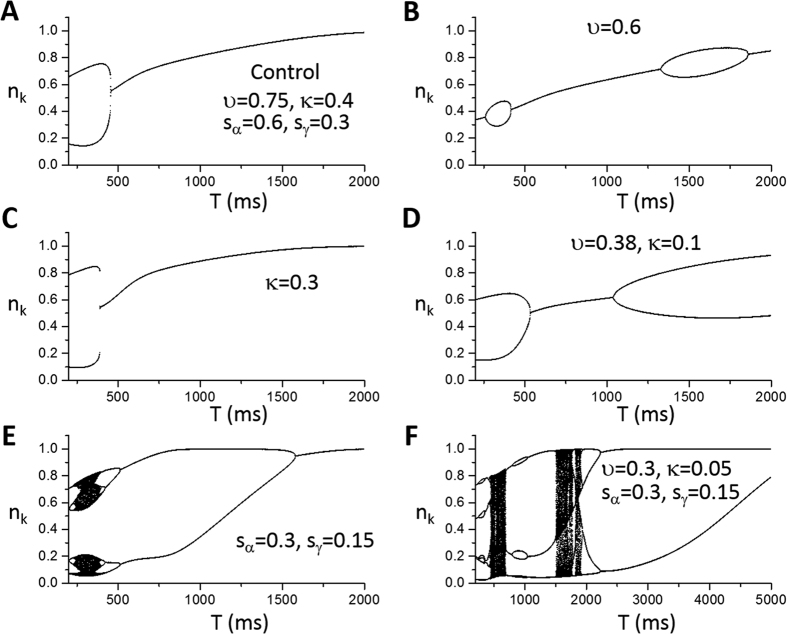
Effects of SERCA pump and fractional SR Ca^2+^ release on dynamics. Shown in each panel is the number of sparks (*n*_k_) versus pacing period (*T*) from the iterated map model (Eqs 7 and 8). (**A**) A control set of parameters. The parameters for the *α* and *γ* functions are the same as for the red curve in Fig. 3B, i.e., *l*_α_ = 3.5, *s*_α_ = 0.6, *l*_γ_ = 3.5, *s*_γ_ = 0.3, *α*_0_ = 1, and *γ*_0_ = 1. The parameters for the *β* function are: *T*_β_ = 500 ms and *τ*_β_ = 75 ms. The parameters for the *h* function are: *υ* = 0.75, *κ* = 0.4, and *σ* = 0.0008. No spark amplitude restitution was presence, i.e., *r*(*T*) = 1, which corresponds to *τ*_r_ = 0. (**B**) Reduced *υ* (*υ* = 0.6). (**C**) Reduced *κ* (*κ* = 0.3). (**D**) Reduced *υ* and *κ* (*υ* = 0.38 and *κ* = 0.1). (**E**) Steepened FCR curve (*s*_α_ = 0.3 and *s*_γ_ = 0.15). (**F**) Reduced *υ*, *κ*, and steepened FCR curve (*υ* = 0.3, *κ* = 0.05, *s*_α_ = 0.3, and *s*_γ_ = 0.15).

**Figure 6:**
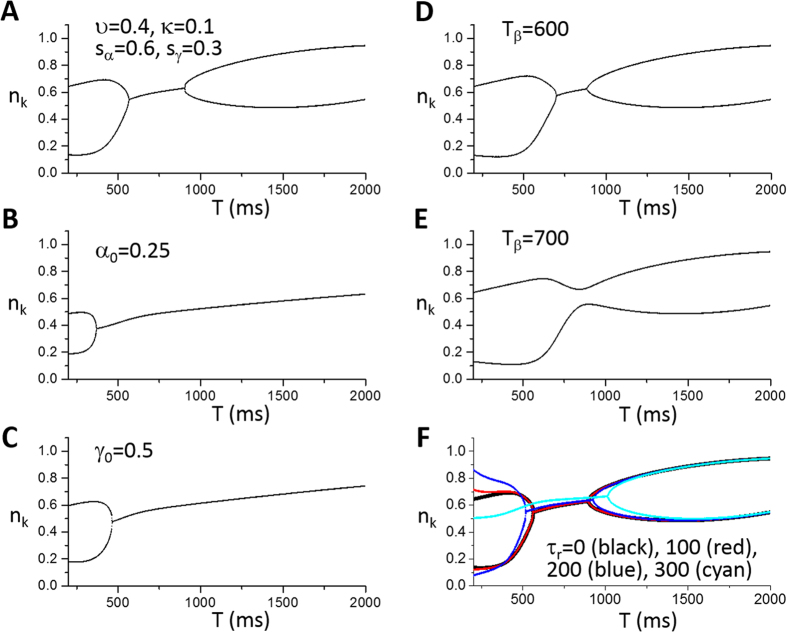
Effects of spark properties on dynamics. Shown in each panel is the number of sparks (*n*_k_) versus pacing period (*T*) from the iterated map model (Eqs 7 and 8). (**A**) Control, which was modified from Fig. 5A by changing *υ* and *κ* (*υ* = 0.4 and *κ* = 0.1). (**B**) Reduced primary spark probability (*α*_0_ = 0.25). (**C**). Reduced recruitment (*γ*_0_ = 0.5). (**D**) *T*_β_ = 600 ms. (**E**) *T*_β_ = 700 ms. (**F**) In the presence of spark amplitude restitution for *τ*_r_ = 0 ms (black, the same as A), 100 ms (red), 200 ms (blue), and 300 ms (cyan).

**Figure 7:**
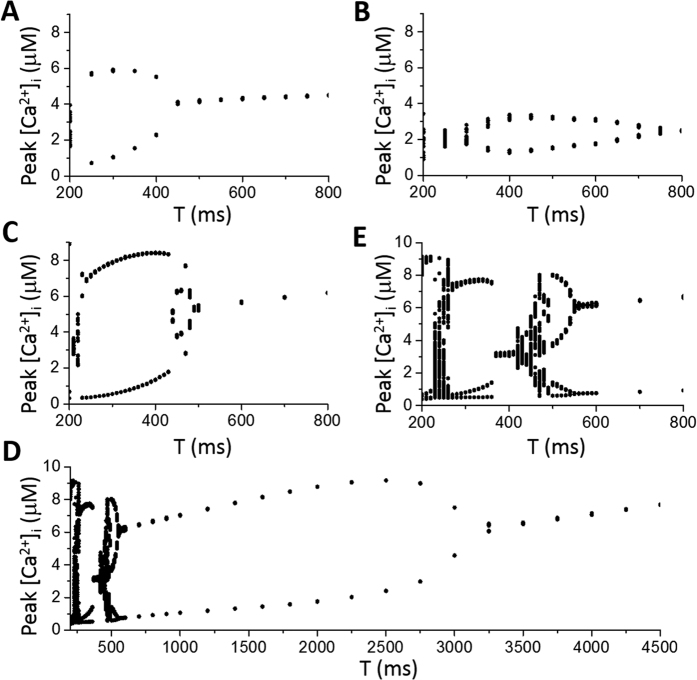
Effects of SERCA pump on dynamics in a detailed model of Ca^2+^ cycling. Shown in each panel is the peak cytosolic Ca^2+^ concentration ([Ca^2+^]_i_) versus pacing period (*T*). For each *T*, the cell was paced with a total of 200 beats and the peak [Ca^2+^]_I_ of the last 100 beats were plotted. (**A**) Control. The control conditions are as in Nivala *et al*.^34^ with [Na]_i_ clamped to 12 mM. (**B**) 50% reduction of maximum SERCA activity (*v*_up_). (**C**) 50% reduction of *K*_up_. (**D**) 50% reduction of both *v*_up_ and *K*_up_. (**E**) An expanded view of D.

For the experiments from Eisner’s group^11^,^12^,^13^,^14^, they showed that dyssynchronous Ca^2+^ release and miniwaves played important roles in the genesis of alternans, which indicates that spark recruitment (the *γ* factor) is important for alternans. Although a steep FCR curve was indicated to be responsible for alternans, it is unclear why dyssynchronous Ca^2+^ release and spark recruitment are also needed. On the other hand, in the 3R theory the roles of dyssynchronous Ca^2+^ release (corresponding to the *α* factor) and miniwaves (corresponding to the *γ* factor) are clear. However, since alternans in these experiments occurred at slow pacing rates (0.5 Hz), thus, it was likely that the RyRs had already or mostly recovered by the beginning of each beat. Alternans via the 3R theory requires a high probability of CRU refractoriness (see Fig. 4B) and thus it cannot satisfactorily explain this set of experiments. The new theory shows that spark recruitment contributes to the steepness of the FCR function. Moreover, it also shows that CRU refractoriness, if there is any, will also synergistically promote alternans (Fig. 6D,E). Therefore, these experiments can now be well explained by the unified theory.

In regards to the experiments showing that DCL alternans is not required for Ca^2+^ alternans^23^,^24^,^25^, the theory shows that this situation can indeed occur when 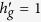, a case in which the amount of Ca^2+^ released by the SR is taken back into the SR. Under these conditions, the two mechanisms are uncoupled and the 3R theory is solely responsible for the mechanism of alternans. However, 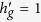 is a very stringent condition, and how this condition can be satisfied in a real cell remains unclear. Specifically, during alternans, the amount of Ca^2+^ released from the SR is also alternating. Since both NCX and SERCA depend nonlinearly on the cytosolic Ca^2+^ concentration, it will be nontrivial for the two pumps to balance in a way such that the SERCA pump uptakes the exact amount of Ca^2+^ released from the SR in the same beat. Therefore, although the 3R theory alone can explain the mechanism of alternans, a robust physiological mechanism still needs to be elucidated to understand how a constant DCL can be maintained during alternans.

However, no experiments have been carried out in a single cell or even in a single species to distinctly demonstrate the two mechanisms. The experiments from Eisner’s group supporting the steep FCR mediated mechanism were done in rat myocytes^11^,^12^,^13^,^14^, the experiments supporting the refractoriness mediated mechanism (namely the 3R theory) were done either in cat^24^ or rabbit^23^,^25^ myocytes. One potential concern would be: are the mechanisms of Ca^2+^ alternans species dependent? First, our simulations using a detailed Ca^2+^ cycling model in our previous study^34^ and the present study show that both mechanisms can occur in the same cell under different Ca^2+^ uptake conditions. Second, there is some experimental evidence for the universality of the two mechanisms. For example, in the study by Xie *et al*.^19^, increased slope of the FCR function and reduced slope of the Ca^2+^ uptake function were demonstrated to promote Ca^2+^ alternans in rabbit ventricular myocytes. Increased refractoriness promoting Ca^2+^ alternans or decreased refractoriness suppressing Ca^2+^ alternans was demonstrated in genetically modified mouse ventricular myocytes^33^,^51^ whose electrophysiological properties share many similarities with those of rat ventricular myocytes. Therefore, we believe that the two mechanisms of Ca^2+^ alternans can be demonstrated in a single cell or a single species by carefully designed experiments to support the unification of the two theories.

However, an iterated map is a low-dimensional mathematical representation of a real system, and when the dynamics of the real system is intrinsically high-dimensional, the iterated map approach may not accurately or even correctly describe the dynamics. For example, Ca^2+^ alternans can be spatially discordant^52^,^53^,^54^, and under these conditions, the low-dimensional description may be problematic. Other spark properties, such as spark duration^42^,^55^,^56^,^57^, may also play important roles, which will need to be taken into account by the theory. Nevertheless, the unified theory can provide a consistent interpretation to the seemly contradict experimental observations and novel mechanistic insights into the mechanisms of Ca^2+^ alternans in ventricular myocytes.

In conclusion, the iterated map model developed in this study provides a cohesive theory for Ca^2+^ alternans in ventricular myocytes, which unifies the two mechanisms of Ca^2+^ alternans into a single theoretical framework. Alternans can be caused by a steep FCR curve combined with a reduced slope of the SR Ca^2+^ uptake function in the absence of refractoriness, or caused by the 3R’s (randomness, recruitment, and refractoriness) when DCL remains constant from beat to beat. In general, the slopes of the FCR and the SR Ca^2+^ uptake functions work in synergy with the 3R’s to promote Ca^2+^ alternans and other complex dynamics in ventricular myocytes. The unification of the two mechanisms of Ca^2+^ alternans can provide novel insight into the identification of therapeutic targets for suppressing Ca^2+^ alternans. For example, reducing the slope of the uptake function (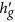) or the coupling of CRUs (*γ*) can suppress both mechanisms of alternans, while reducing the refractory period can only suppress alternans caused by one of the two mechanisms. Suppressing Ca^2+^ alternans by reducing the slope of the uptake function has been demonstrated by experiments of SERCA2a overexpression^19^,^58^, supporting our theoretical predictions.

## Additional Information

**How to cite this article**: Qu, Z. *et al*. A unified theory of calcium alternans in ventricular myocytes. *Sci. Rep.* **6**, 35625; doi: 10.1038/srep35625 (2016).
